# Discovery of Novel *N*-Substituted Prolinamido Indazoles as Potent Rho Kinase Inhibitors and Vasorelaxation Agents

**DOI:** 10.3390/molecules22101766

**Published:** 2017-10-19

**Authors:** Yangyang Yao, Renze Li, Xiaoyu Liu, Feilong Yang, Ying Yang, Xiaoyu Li, Xiang Shi, Tianyi Yuan, Lianhua Fang, Guanhua Du, Xiaozhen Jiao, Ping Xie

**Affiliations:** 1 State Key Laboratory of Bioactive Substances and Functions of Natural Medicines, Beijing Key Laboratory of Active Substances Discovery and Druggability Evaluation, Institute of Materia Medica, Chinese Academy of Medical Sciences and Peking Union Medical College, Beijing 100050, China; yao0943017@163.com (Y.Y.); renze.li@open01.com (R.L.); lxya@imm.ac.cn (X.L.); yangfeilong@imm.ac.cn (F.Y.); yangying@imm.ac.cn (Y.Y.); pharmli@imm.ac.cn (X.L.); xiangshi@imm.ac.cn (X.S.); 2 State Key Laboratory of Bioactive Substances and Functions of Natural Medicines, Beijing Key Laboratory of Drug Targets Identification and Drug Screening, Institute of Materia Medica, Chinese Academy of Medical Sciences and Peking Union Medical College, Beijing 100050, China; yuantianyi@imm.ac.cn (T.Y.); fanglh@imm.ac.cn (L.F.); dugh@imm.ac.cn (G.D.)

**Keywords:** Rho kinase, inhibitor, *N*-substituted prolinamido indazoles, vasorelaxant, SAR

## Abstract

Inhibitors of Rho kinase (ROCK) have potential therapeutic applicability in a wide range of diseases, such as hypertension, stroke, asthma and glaucoma. In a previous article, we described the lead discovery of DL0805, a new ROCK I inhibitor, showing potent inhibitory activity (IC_50_ 6.7 μM). Herein, we present the lead optimization of compound DL0805, resulting in the discovery of 24- and 39-fold more-active analogues **4a** (IC_50_ 0.27 μM) and **4b** (IC_50_ 0.17 μM), among other active analogues. Moreover, ex-vivo studies demonstrated that **4a** and **4b** exhibited comparable vasorelaxant activity to the approved drug fasudil in rat aortic rings. The research of a preliminary structure–activity relationship (SAR) indicated that the target compounds containing a β-proline moiety have improved activity against ROCK I relative to analogues bearing an α-proline moiety, and among the series of the derivatives with a β-proline-derived indazole scaffold, the inhibitory activity of the target compounds with a benzyl substituent is superior to those with a benzoyl substituent.

## 1. Introduction

Arterial hypertension is a long-term medical condition in which the blood pressure in the arteries is persistently elevated. Hypertension already affects 1.1 billion people worldwide, leading to coronary artery disease, stroke, heart failure, peripheral vascular disease, vision loss and chronic kidney disease. Despite the development of approaches for the prevention and control of raised blood pressure, there remains a significant need for the discovery of novel antihypertensive agents.

Rho kinase (Rho-associated coiled-coil-containing protein kinase (ROCK)) belongs to a family of serine/threonine protein kinases and acts as a major downstream effector of Rho A [1]. It plays an important role in regulating a variety of cellular functions such as smooth-muscle-cell contraction, cell adhesion, migration and proliferation [2]. ROCK contains two isoforms, ROCK I and ROCK II, which share approximately 60% overall amino acid sequence identity and 92% identity within the kinase domain [3]. However, the expression of the two isoforms has different tissue specificity. ROCK II shows high expression in brain tissue, while ROCK I is preferentially expressed in the tissues of the pancreas, liver, lung and heart [3]. Accumulating evidence shows that the dysregulation of ROCK activity or expression is associated with various diseases, including hypertension [4], stroke [5], asthma [6,7], cancer [8,9] and glaucoma [10,11,12], which indicates that ROCK is a potential novel target for drug development.

Over the past two decades, ROCK has aroused extensive interest, and numerous ROCK inhibitors have been developed from a variety of distinct scaffolds including isoquinoline [13], quinazoline [14], indazole [15,16,17,18,19,20], benzimidazole [21,22], benzothiazole [23], quinazolinone [24,25], diaminopyrazine [26], benzamide [27], chroman-3-amide [28] and urea [29]. Usually, ROCK II inhibitors were widely applied in the central nervous system (CNS) to cure stroke, Alzheimer’s disease and other diseases, and ROCK I inhibitors showed therapeutic potential for the treatment of hypertension [30]. To date, only two ROCK inhibitors, fasudil (approved in 1995 for the treatment of cerebral vasopasm in Japan) and ripasudil (approved in 2014 for treatment of glaucoma in Japan) have been approved for clinical use. The narrow therapeutic window restricts the application of the existing ROCK inhibitors, leading to the development of novel ROCK inhibitors. Our group has been focused on the discovery of novel Rho kinase inhibitors for the treatment of hypertension.

**Figure 1:**
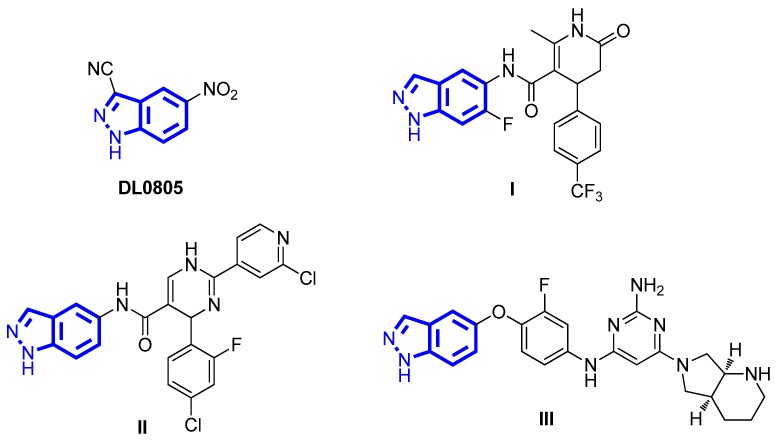
The structures of screened lead compound DL0805, I, II and III.

5-Nitro-1*H*-indazole-3-carbonitrile (DL0805, **1**) (Figure 1) is a new ROCK inhibitor with an IC_50_ value of 6.7 μM against ROCK I, which was discovered by high-throughput screening (HTS) [31]. Previous literature had disclosed the development of ROCK I inhibitors with an indazole scaffold attached to a rigid aromatic heterocycle linking structure (I, II, III) (Figure 1) [32,33,34]. These studies highlighted the importance of the indazole for ROCK I activity. Herein, we report the design, synthesis and the structure–activity relationship (SAR) research of a series of novel *N*-substituted prolinamido indazoles as ROCK I inhibitors, and their vasorelaxant activity evaluation based on the DL0805 template.

## 2. Results and Discussion

### 2.1. Molecular Design

**Figure 2:**
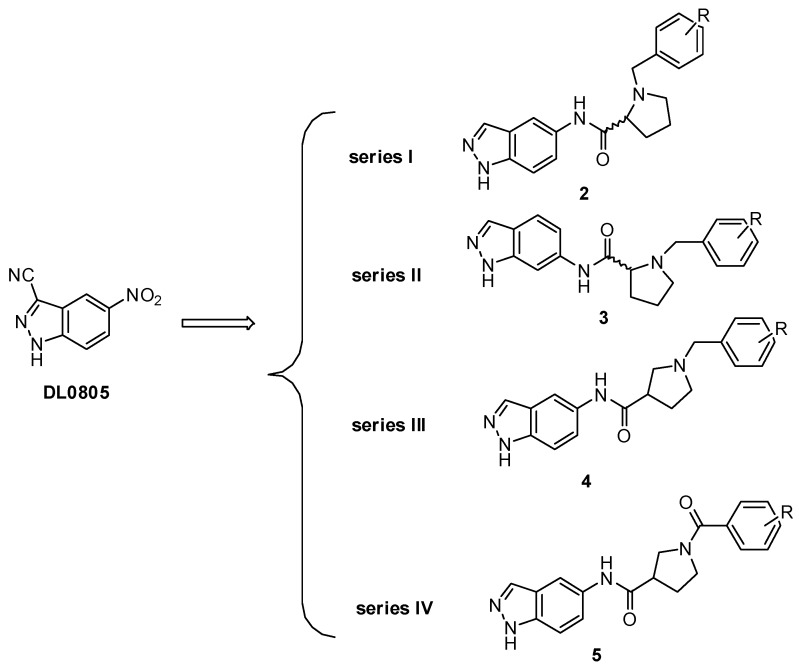
Molecular design based on the template compound DL0805.

In order to discover potent ROCK I inhibitors, we further optimized the structure of the DL0805 template. The attractive indazole core of DL0805 was preserved, owing to the frequent presence of this scaffold in ROCK I inhibitors. Considering the potential genotoxic hazard of the NO_2_ group, we conceived of the novel *N*-substituted prolinamido indazole ROCK I inhibitors, wherein the NO_2_ group can be replaced with a flexible *N*-substituted prolinamido group instead of the substituted rigid heteroaromatic ring described in the literature (e.g., I, II, III) (Figure 1). Four related series of *N*-substituted prolinamido indazoles were designed and are listed in Figure 2 (**2**, **3**, **4**, **5**).

### 2.2. Chemistry

**Scheme 1:**
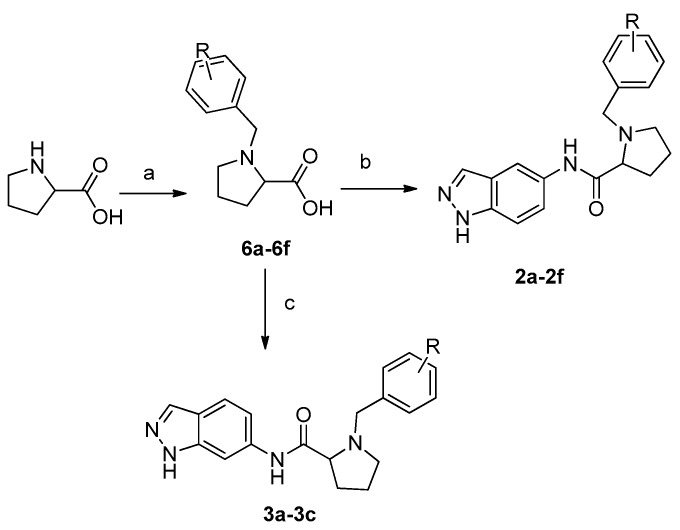
Synthesis of compounds **2a**–**2f** and **3a**–**3c**. Reagents and conditions: (a) R-Br, KOH, isopropyl alcohol, 40 °C, 12 h; (b) 5-aminoindazole, EDCI, DMF, 80 °C, 3 h; (c) 6-aminoindazole, EDCI, DMF, 80 °C, 3 h.

A concise route was developed to synthesize compounds **2a**–**2f** and **3a**–**3c** using racemic or D/L-proline as the starting material (Scheme 1). Alkylation of proline provided the intermediates **6a**–**6f**. Initially, amidation of **6a** with 5-aminoindazole employing EDCI in CH_2_Cl_2_ at room temperature gave the target compound **2a** only in low yield because of **6a**’s solubility. When we changed to use DMF as reaction solvent and raised the reaction temperature to 80 °C, target compound **2a** was obtained in moderate yield. Thus, this reaction condition was used to synthesize the target compounds **2b**–**2f**. Analogues **3a**–**3c** were similarly prepared by coupling the intermediates **6a**–**6c** with 6-aminoindazole.

**Scheme 2:**
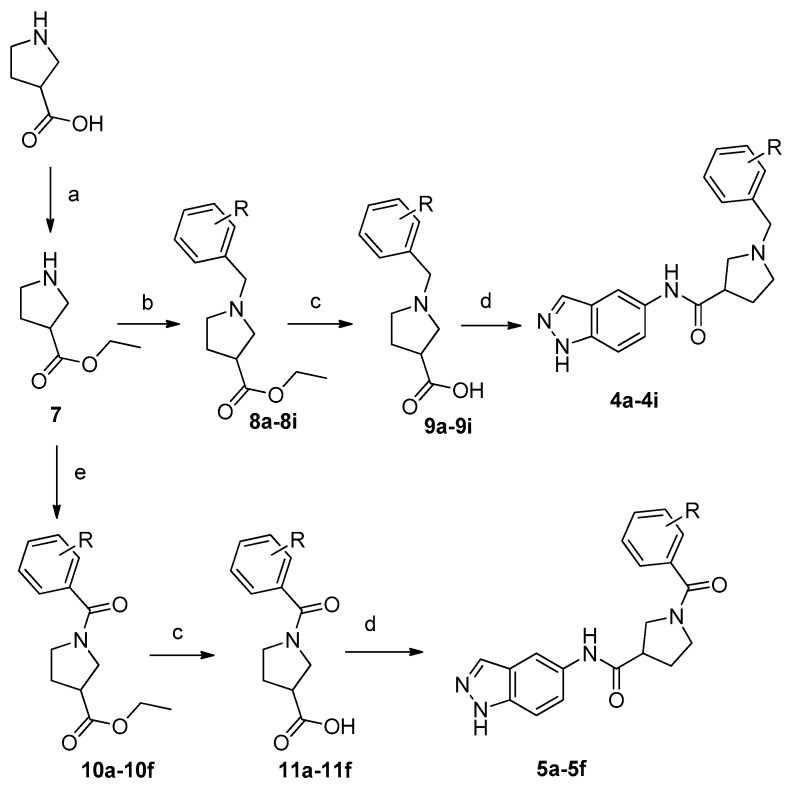
Synthesis of compounds **4a**–**4i** and **5a**–**5f**. Reagents and conditions: (a) SOCl_2_, ethyl alcohol, 0 to 40 °C, 10 h; (b) R-Br, Et_3_N, CH_2_Cl_2_, reflux, 4 h; (c) 4 M NaOH, ethyl alcohol, room temperature, 1 h; (d) 5-aminoindazole, EDCI, DMF, 80 °C, 7 h; (e) R-benzoyl chloride, Et_3_N, CH_2_Cl_2_, reflux, 4 h.

Next, we embarked on the synthesis of analogue **4** following a similar synthetic route used for preparing compounds **2** and **3**. Unfortunately, an inseparable mixture was observed during the direct alkylation of β-proline. Therefore, new synthetic strategies to access analogues **4** and **5** were performed and described in Scheme 2. The target compounds **4a**–**4i** were synthesized using racemic or D/L-β-proline as the starting material. Esterification of β-proline with SOCl_2_ and ethyl alcohol gave the ester **7**. Subsequently, alkylation of ester **7** with substituted benzyl bromides provided compounds **8a**–**8i**, which were hydrolyzed and then reacted with 5-aminoindazole to produce compounds **4a**–**4i**. Intermediate **7** was also used to prepare the target compounds **5a**–**5f** in an analogous fashion. Acylation of ester **7** with substituted benzoyl chloride afforded compounds **10a**–**10f** that were saponified to **11a**–**11f** and coupled to 5-aminoindazole to furnish the other set of target compounds **5a**–**5f**.

### 2.3. Bioassay Studies

#### 2.3.1. ROCK I Inhibitory Activity Evaluation

The twenty-four target compounds were initially evaluated for their percentage inhibition against ROCK I with a ROCK I assay kit (CY-1160, Cyclex, Nagoya, Japan) at 20 μM (Table 1). The nine compounds with significant inhibition against ROCK I were further evaluated in full concentration–response plots to determine their IC_50_ values (Table 2). The data illustrate some clear SAR trends. Firstly, the target compounds of series III (**4**) and IV (**5**) containing a β-proline moiety have improved activity against ROCK I relative to analogue series I (**2**) and II (**3**) bearing an α-proline moiety. This implied that a near-linear molecule probably had better combination with ROCK I than an “angular”-shaped molecule. Secondly, among the series of the derivatives with a β-proline-derived indazole scaffold, the inhibitory activity of the target compounds (series III) with a benzyl substituent (**4**) was superior to those with a benzoyl substituent (series IV **5**). This suggested that the free rotation of a single bond was probably beneficial to the ROCK I inhibitory activity. Thirdly, the data of series III compounds showed that the substituent group had some influence on the activity (CH_3_ > H > Br > OCH_3_ > F > NO_2_, CN). This implied that the activity was probably affected by multiple factors, and the volume factor may be important for the activity. In addition, the data also revealed the (*S*)-enantiomer had a higher activity than the (*R*)-enantiomer (**4i** versus **4h**, respectively) with the (1*H*-indazole-5-yl)-pyrrolidine-3-carboxamide scaffold. In contrast, the (*R*)- and (*S*)-enantiomers had a similar activity (**2b** versus **2c**; **3b** versus **3c**) in the (1*H*-indazole-5-yl)-pyrrolidine-2-carboxamide and the (1*H*-indazole-6-yl)-pyrrolidine-3-carboxamide scaffolds.

#### 2.3.2. Vasorelaxant Activity Evaluation

Abnormalities in the Rho/ROCK signaling pathway are associated with various cardiovascular diseases, especially hypertension. The inhibition of the Rho/ROCK pathway can cause the vessels to relax [4]. The norepinephrine (NE)- or potassium chloride (KCl)-induced model of the rat aortic ring can trigger sustained vessel contraction and is usually used to evaluate vasorelaxant activity. DL0805 has shown vasorelaxant activity [31]. Therefore, the nine compounds with significant inhibition against ROCK I were further tested for their vasorelaxant activity in rat aortic rings in both the high-potassium and NE models [35] (Table 3). Compounds **4a**, **4b** and **4c** showed low micromolar EC_50_ values in both vasorelaxant assays. The potent active compound **4b** with excellent activity has been further evaluated [35], and further pharmacokinetic and safety evaluations are in progress.

### 2.4. Molecular Docking Studies

**Figure 3:**
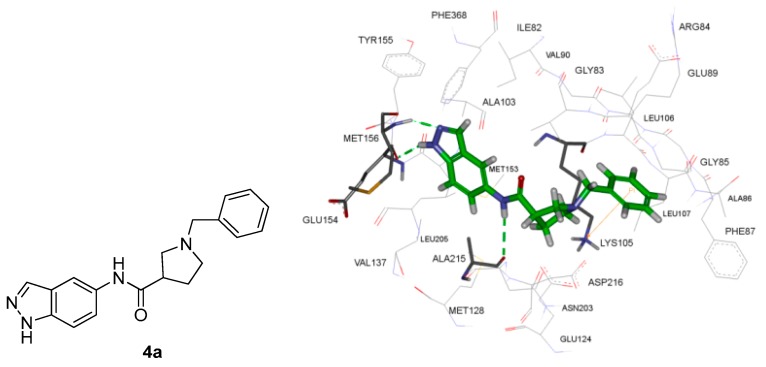

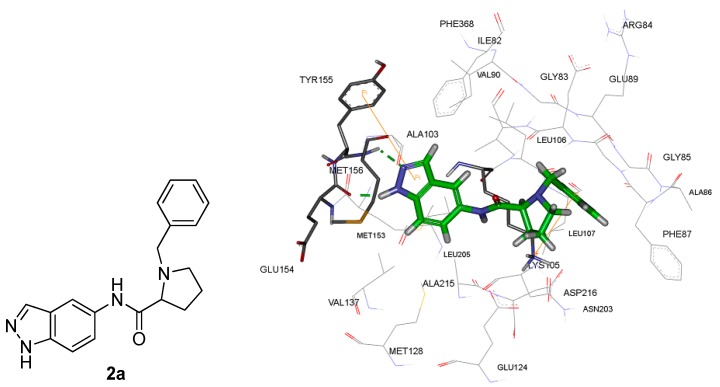
The 2D structure of compounds **4a** and **2a**, and 3D view of the key interactions between **4a**, **2a** and the ATP binding pocket of ROCK I. Docking study was performed with the ROCK I structure obtained from the Protein Data Bank (PDB code: 3NDM). Green dashed lines represent hydrogen-bonding interactions and orange lines represent pi–cation interaction.

To identify the possible binding modes of our inhibitors, molecular docking of compound **4a** was performed to elucidate key interactions within the active site of ROCK I. As shown in Figure 3, docking of compound **4a** into the binding site of Rho kinase indicates two key hydrogen-bond interactions between the N and NH in the indazole ring and Met 156, respectively. The amide NH is predicted to form a hydrogen bond with Ala 215. Furthermore, a pi–cation interaction between the terminal phenyl ring and Lys 105 was also observed. In addition, molecular docking of compound **2a** was also performed. In contrast to compound **4a**, two key hydrogen-bond interactions between the N and NH in the indazole ring and Met 156 were still maintained. However, the hydrogen-bond interaction between the amide NH with Ala 215 was not observed. The result is in agreement with the activity result, that the activities of series III and IV were superior to those of series I and II.

## 3. Materials and Methods

All melting points were obtained on a Yanaco melting-point apparatus and were uncorrected. ESI mass spectra were performed on the Thermo Exactive Plus LC–MS spectrometer. Nuclear magnetic resonance (NMR) spectra were recorded on a Bruker 300 or 400 MHz NMR spectrometer with TMS as an internal standard. Chemical shifts (δ values) and coupling constants (*J* values) were given in ppm and Hz, respectively. Column chromatography was performed with silica gel (160–200 mesh, Qingdao Haiyang Chemical, Qingdao, China). Unless otherwise noted, reagents and solvents were purchased from Acros Chemical Co, (Geel, Belgium) or other commercial providers and used without further purification. 

### 3.1. Chemistry

#### 3.1.1. General Procedure for the Preparation of Compounds **6**

To a solution of proline (2.0 g, 17 mmol) and KOH (2.85 g, 51 mmol) in *i-*PrOH (50 mL), 4-substituted benzyl derivative (21 mmol) was added at 40 °C. Afterwards, the mixture was stirred for 8 h at 40 °C and then the reaction mixture was cooled to room temperature. 6 M HCl was added to adjust the pH value of the mixture to 4–5 and then CHCl_3_ (50 mL) was added. The mixture was stirred for 12 h, followed by filtration and evaporation in vacuo. The residue was purified by recrystallization in acetone at 0 °C to give **6** (about 90% yield).

*1-Benzylpyrrolidine-2-carboxylic acid* (**6a**): yield: 86%; white solid; ^1^H-NMR (400 MHz, CD_3_OD): δ 7.53 (m, 2H), 7.47 (m, 3H), 4.53 (d, 1H, *J* = 12.0 Hz), 4.28 (d, *J* = 12.8 Hz, 1H), 4.14 (dd, *J*_1_ = 10.4 Hz, *J*_2_ = 7.2 Hz, 1H), 3.52 (m, 1H), 3.27 (m, 1H), 2.54 (m, 1H), 2.14 (m, 2H), 2.20 (m, 1H).

*(R)-1-Benzylpyrrolidine-2-carboxylic acid* (**6b**): yield: 90%; white solid; [α]${}_{D}^{27}$ = +25.3 (c 0.92, MeOH); ^1^H-NMR (400 MHz, CD_3_OD): δ 7.53 (m, 2H), 7.49 (m, 3H), 4.57 (d, 1H, *J* =10.8 Hz), 4.37 (t, *J* = 8.8 Hz, 1H), 4.31 (d, *J* = 12.8 Hz, 1H), 3.51 (m, 1H), 3.35 (m, 1H), 2.61 (m, 1H), 2.18 (m, 2H), 2.00 (m, 1H).

*(S)-1-Benzylpyrrolidine-2-carboxylic acid* (**6c**): yield: 92%; white solid; [α]${}_{D}^{26}$ = −26.9 (c 0.9, MeOH); ^1^H-NMR (400 MHz, CD_3_OD): δ 7.51 (m, 2H), 7.45 (m, 3H), 4.51 (d, 1H, *J* = 12.8 Hz), 4.26 (d, *J* = 12.4 Hz, 1H), 4.10 (m, 1H), 3.49 (m, 1H), 3.26 (m, 1H), 2.52 (m, 1H), 2.13 (m, 2H), 1.96 (m, 1H).

*1-(4-Methylbenzyl)pyrrolidine-2-carboxylic acid* (**6d**): yield: 84%; white solid; ^1^H-NMR (400 MHz, DMSO-*d*_6_): δ 7.33 (d, *J* = 7.6 Hz, 2H), 7.19 (d, *J* = 7.6 Hz, 2H), 4.21 (d, *J* = 12.8 Hz, 1H), 4.00 (d, *J* = 12.8 Hz, 1H), 3.76 (t, *J* = 7.2 Hz, 1H), 3.25 (m, 1H), 2.91 (q, *J* = 9.2 Hz, 1H), 2.29 (s, 4H), 2.23 (m, 1H), 1.91 (m, 2H), 1.84 (m, 1H), 1.77 (m, 1H). ^13^C-NMR (150 MHz, DMSO): δ 170.6, 138.0, 130.1, 129.8, 129.1, 65.7, 56.7, 53.3, 28.1, 22.2, 20.8.

*(R)-1-(4-Methylbenzyl)pyrrolidine-2-carboxylic acid* (**6e**): yield: 89%; white solid; [α]${}_{D}^{28}$ = +27.3 (c 0.92, MeOH); ^1^H-NMR (400 MHz, DMSO-*d*_6_): δ 7.31 (d, *J* = 7.6 Hz, 2H), 7.18 (d, *J* = 7.6 Hz, 2H), 4.13 (d, *J* = 12.8 Hz, 1H), 3.89 (d, *J* = 12.8 Hz, 1H), 3.52 (dd, *J*_1_ = 6.4 Hz, *J*_2_ = 8.4 Hz, 1H), 3.18 (m, 1H), 2.76 (q, *J* = 9.2 Hz, 1H), 2.30 (s, 4H), 2.16 (dd, *J*_1_ = 8.8 Hz, *J*_2_ = 12.4 Hz, 1H), 1.89 (m, 2H), 1.84 (m, 1H), 1.74 (m, 1H). ^13^C-NMR (150 MHz, DMSO): δ 170.9, 137.8, 130.5, 130.0, 129.0, 65.8, 56.8, 53.2, 28.3, 22.4, 20.8.

*(S)-1-(4-Methylbenzyl)pyrrolidine-2-carboxylic acid* (**6f**): yield: 92%; white solid; [α]${}_{D}^{26}$ = −23 (c 0.92, MeOH); ^1^H-NMR (400 MHz, DMSO-*d*_6_): δ 7.38 (d, *J* = 7.6 Hz, 2H), 7.22 (d, *J* = 7.6 Hz, 2H), 4.32 (d, *J* = 12.8 Hz, 1H), 4.13 (d, *J* = 12.8 Hz, 1H), 3.97 (t, *J* = 7.6 Hz, 1H), 3.33 (m, 1H), 3.04 (q, *J* = 10 Hz, 1H), 2.30 (s, 4H), 1.95 (m, 2H), 1.89 (m, 1H), 1.81 (m, 1H), 1.74 (m, 1H). ^13^C-NMR (150 MHz, DMSO): δ 170.2, 138.4, 130.4, 129.1, 129.0, 65.4, 56.7, 53.6, 28.1, 22.1, 20.8.

#### 3.1.2. General Procedure for the Preparation of Compounds **7**

To a solution of ethanol (150 mL), SOCl_2_ (10.34 g, 86.86 mmol) was added at 0 °C. Afterwards, β-proline (5.0 g, 43.43 mmol) was added in several separated portions at 0 °C. The mixture was stirred for 10 h at 40 °C and then evaporated in vacuo. The residue was used immediately without further purification.

#### 3.1.3. General Procedure for the Preparation of Compounds **8** or **10**

To a solution of **7** (400 mg) in dry CH_2_Cl_2_ (8 mL), Et_3_N (849 mg, 8.39 mmol) and 4-substituted benzyl or benzoyl derivative (3.36 mmol) were added at room temperature. Afterwards, the mixture was refluxed for 4 h and then evaporated in vacuo. The residue was purified by column chromatography on silica gel (10–20% EtOAc in CH_2_Cl_2_) to give **8** or **10** (about 80% yield).

*Ethyl 1-benzylpyrrolidine-2-carboxylate* (**8a**): yield: 79%; slightly yellow oil; ^1^H-NMR (CDCl_3_, 400 MHz): δ 7.27–7.44 (m, 5H), 4.15 (q, *J* = 7.2 Hz, 2H), 3.84 (s, 2H), 3.12 (m, 2H), 2.99 (m, 1H), 2.83 (m, 1H), 2.68 (m, 1H), 2.23 (m, 1H), 2.16 (m, 1H), 1.25 (t, *J* = 7.2 Hz, 3H).

*Ethyl 1-(4-methylbenzyl)pyrrolidine-2-carboxylate* (**8b**): yield: 73%; slightly yellow oil; ^1^H-NMR (CDCl_3_, 400 MHz): δ 7.21 (d, *J* = 7.6 Hz, 2H), 7.12 (d, *J* = 7.6 Hz, 2H), 4.04 (q, *J* = 7.2 Hz, 2H), 3.61 (s, 2H), 3.02 (p, *J* = 6.8 Hz, 1H), 2.94 (t, *J* = 8.8 Hz, 1H), 2.73 (m, 1H), 2.61 (t, *J* = 8.0 Hz, 1H), 2.51 (d, *J* = 8.0 Hz, 1H), 2.33 (s, 3H), 2.10 (p, *J* = 7.2 Hz, 1H), 1.24 (t, *J* = 7.2 Hz, 3H).

*Ethyl 1-(4-methoxylbenzyl)pyrrolidine-2-carboxylate* (**8c**): yield: 80%; slightly yellow oil; ^1^H-NMR (CDCl_3_, 400 MHz): δ 7.43 (d, *J* = 8.4 Hz, 2H), 6.88 (d, *J* = 8.8 Hz, 2H), 4.13 (q, *J* = 7.2 Hz, 2H), 3.98 (s, 2H), 3.78 (s, 3H), 3.42 (m, 1H), 3.26 (m, 2H), 3.04 (m, 1H), 2.83 (m, 1H), 2.38 (m, 1H), 2.17 (m, 1H), 1.24 (t, *J* = 7.2 Hz, 3H).

*Ethyl 1-(4-bromobenzyl)pyrrolidine-2-carboxylate* (**8d**): yield: 74%; slightly yellow oil; ^1^H-NMR (400 MHz, DMSO): δ 7.49 (d, *J* = 8.0 Hz, 2H), 7.24 (d, *J* = 8.0 Hz, 2H), 4.04 (q, *J* = 7.1 Hz, 2H), 3.54 (m, 2H), 2.98 (p, *J* = 6.8 Hz, 1H), 2.67 (t, *J* = 8.8 Hz, 1H), 2.58 (m, 1H), 2.46 (m, 2H), 1.95 (m, 2H), 1.15 (t, *J* = 7.1 Hz, 3H). ^13^C-NMR (150 MHz, DMSO): δ 174.23, 138.48, 131.02, 130.56, 119.77, 60.07, 58.10, 55.91, 53.07, 41.31, 27.12, 14.09.

*Ethyl 1-(4-nitrobenzyl)pyrrolidine-2-carboxylate* (**8e**): yield: 83%; slightly yellow oil; ^1^H-NMR (500 MHz, CDCl_3_): δ 8.18 (d, *J* = 8.2 Hz, 2H), 7.52 (m, 2H), 4.15 (q, *J* = 7.2 Hz, 2H), 3.74 (s, 2H), 3.27–2.36 (m, 5H), 2.13 (m, 2H), 1.26 (t, *J* = 7.1 Hz, 4H). ^13^C-NMR (100 MHz, CDCl_3_): δ 129.11, 123.52, 60.68, 59.13, 56.58, 53.78, 42.12, 27.71, 14.19. HR–ESI–MS: *m*/*z* = 279.1324 [M + H]^+^, calcd. for C_14_H_19_N_2_O_4_: 279.1339.

*Ethyl 1-(4-cyanobenzyl)pyrrolidine-2-carboxylate* (**8f**): yield: 73%; slightly yellow oil; ^1^H-NMR (400 MHz, CDCl_3_): δ 7.60 (d, *J* = 7.9 Hz, 2H), 7.45 (d, *J* = 7.9 Hz, 2H), 4.14 (q, *J* = 7.1 Hz, 2H), 3.69 (m, 2H), 3.02 (m, 1H), 2.83 (t, *J* = 8.8 Hz, 1H), 2.68 (m, 2H), 2.58 (m, 1H), 2.12 (m, 2H), 1.25 (t, *J* = 7.1 Hz, 3H).^13^C-NMR (100 MHz, CDCl_3_): δ 174.8, 132.1, 129.1, 118.9, 110.8, 109.7, 60.7, 59.4, 56.6, 53.7, 42.1, 27.7, 14.2. HR–ESI–MS: *m*/*z* = 259.1432 [M + H]^+^, calcd. for C_15_H_19_N_2_O_2_: 259.1441.

*Ethyl 1-benzoylpyrrolidine-2-carboxylate* (**10a**): yield: 77%; slightly yellow oil; ^1^H-NMR (400 MHz, CDCl_3_): δ 7.51 (m, 2H), 7.40 (m, 3H), 4.17 (q, *J* = 7.1 Hz, 2H), 3.71 (s, 3H), 3.09 (s, 1H), 2.20 (s, 2H), 2.04 (s, 1H), 1.26 (t, *J* = 7.1 Hz, 3H). ^13^C-NMR (150 MHz, CDCl_3_): δ 169.8, 136.6, 130.0, 128.3, 127.1, 61.1, 14.2. HR–ESI–MS: *m*/*z* = 248.1274 [M + H]^+^, calcd. for C_14_H_18_NO_3_: 248.1281.

*Ethyl 1-(4-methylbenzoyl) pyrrolidine-2-carboxylate* (**10b**): yield: 74%; slightly yellow oil; ^1^H-NMR (500 MHz, CDCl_3_): δ 7.42 (d, *J* = 7.7 Hz, 2H), 7.19 (d, *J* = 7.7 Hz, 2H), 4.16 (s, 2H), 3.76 (m, 3H), 3.07 (s, 1H), 2.37 (s, 3H), 2.19 (s, 2H), 1.26 (t, *J* = 7.1 Hz, 3H). ^13^C-NMR (150 MHz, CDCl_3_): δ 173.0, 172.4, 169.8, 140.2, 133.6, 130.0, 129.0, 128.9, 127.2, 61.0, 51.4, 48.8, 48.5, 45.5, 43. 8, 41.9, 29.4, 27.9, 21.4, 14.1.

*Ethyl 1-(4-methoxylbenzoyl)pyrrolidine-2-carboxylate* (**10c**): yield: 87%; slightly yellow oil; ^1^H-NMR (500 MHz, CDCl_3_): δ 7.51 (d, *J* = 7.9 Hz, 2H), 6.90 (d, *J* = 8.1 Hz, 2H), 4.17 (q, *J* = 7.2 Hz, 2H), 3.83 (s, 3H), 3.66 (m, 3H), 3.08 (s, 1H), 2.19 (d, *J* = 7.4 Hz, 2H), 1.79 (s, 1H), 1.26 (t, *J* = 7.1 Hz, 3H).

*Ethyl 1-(4-bromobenzoyl)pyrrolidine-2-carboxylate* (**10d**): yield: 74%; slightly yellow oil; ^1^H-NMR (400 MHz, DMSO): δ 7.49 (d, *J* = 8.0 Hz, 2H), 7.24 (d, *J* = 8.0 Hz, 2H), 4.04 (q, *J* = 7.1 Hz, 2H), 3.54 (m, 2H), 2.98 (p, *J* = 6.8 Hz, 1H), 2.67 (t, *J* = 8.8 Hz, 1H), 2.58 (m, 1H), 2.46 (m, 2H), 1.95 (m, 2H), 1.15 (t, *J* = 7.1 Hz, 3H). ^13^C-NMR (150 MHz, DMSO): δ 174.2, 138.5, 131.0, 130.6, 119.8, 60.0, 58.1, 55.9, 53.1, 41.3, 27.1, 14.1.

*Ethyl 1-(4-nitrobenzoyl)pyrrolidine-2-carboxylate* (**10e**): yield: 74%; slightly yellow oil; ^1^H-NMR (400 MHz, CDCl_3_): δ 8.27 (dd, *J* = 8.6, 3.1 Hz, 2H), 7.68 (dd, *J* = 8.6, 2.5 Hz, 2H), 4.18 (dq, *J* = 21.6, 7.2 Hz, 2H), 3.92 (d, *J* = 7.1 Hz, 1H), 3.41–3.84 (m, 3H), 3.13 (dt, *J* = 38.4, 7.1 Hz, 1H), 2.23 (ddt, *J* = 32.8, 13.1, 6.7 Hz, 2H), 1.27 (m, 3H). ^13^C-NMR (150 MHz, CDCl3): δ 172.8, 172.1, 167.5, 167.3, 148.6, 142.5, 142.5, 128.2, 128.16, 123.8, 123.708, 61.3, 61.2, 51.0, 48.7, 48.5, 45.6, 43.7, 41.8, 29.4, 27.8, 14.2, 14.1.HR–ESI–MS: *m*/*z* = 293.1120 [M + H]^+^, calcd. for C_14_H_1__7_N_2_O_5_: 293.1132.

*Ethyl 1-(4-chlorobenzoyl)pyrrolidine-2-carboxylate* (**10f**): yield: 74%; slightly yellow oil; ^1^H-NMR (600 MHz, CDCl_3_): δ 7.47 (d, *J* = 8.0 Hz, 2H), 7.38 (dd, *J* = 8.4, 4.0 Hz, 2H), 4.23–4.07 (m, 2H), 3.89 (m, 1H), 3.81–3.41 (m, 3H), 3.09 (m, 1H), 2.35–2.05 (m, 2H), 1.26 (m, 3H). ^13^C-NMR (150 MHz, CDCl_3_): δ 172.9, 172.3, 168.7, 168.5, 136.1, 134.9, 134.9, 128.7, 128.7, 128.6, 128. 6, 61.2, 61.1, 51.3, 48.7, 48.6, 45.6, 43.8, 41.8, 29.4, 27.8, 14.2, 14.1. HR–ESI–MS: *m*/*z* = 282.0877 [M + H]^+^, calcd. for C_14_H_17_NO_3_Cl: 282.0892.

#### 3.1.4. General Procedure for the Preparation of Compounds **9** or **11**

To a solution of **8** or **10** (0.775 mmol) in EtOH (3 mL), 4 M NaOH (0.7 mL, 2.8 mmol) was added at room temperature. Afterwards, the mixture was stirred for 1 h and then evaporated in vacuo. After being diluted with water and extracted with EtOAc, the pH of the aqueous phase was adjusted to 1–2 with 6 M HCl. The mixture was extracted with *n*-BuOH and then the organic phase was washed with saturated NaCl. The organic phase was dried over Na_2_SO_4_ and evaporated in vacuo. The residue was used immediately without further purification.

#### 3.1.5. General Procedure for the Preparation of Target Compounds **2a**–**2f**; **3a**–**3c**; **4a**–**4i**; **5a**–**5f**

To a solution of intermediate **9**, **11** or **6** (0.39 mmol) and 5- or 6-aminoindazole (0.47 mmol) in dry DMF (3 mL), EDCI (90 mg, 0.47 mmol) was added at r.t. Afterwards, the mixture was stirred for 7 h at 80 °C and then the reaction mixture was cooled to room temperature. The mixture was evaporated in vacuo and the residue was purified by column chromatography on silica gel (2%–10% CH_3_OH in CH_2_Cl_2_) to give the target compounds.

*1-Benzyl-N-(1H-indazol-5-yl)pyrrolidine-2-carboxamide* (**2a**): yield: 62%; white solid; m.p.: 200–201 °C; ^1^H-NMR (300 MHz, CDCl_3_): δ 12.96 (s, 1H), 9.67 (s, 1H), 8.08 (s, 1H), 7.99 (s, 1H), 7.41 (m, 4H), 7.30 (t, *J* = 7.2 Hz, 2H), 7.22 (d, *J* = 6.9 Hz, 1H), 3.85 (d, *J* = 12.9 Hz, 1H), 3.59 (d, *J* = 13.2 Hz, 1H), 3.24 (dd, *J*_1_ = 4.5 Hz; *J*_2_ = 8.7 Hz, 1H), 3.03 (m, 1H), 2.39 (q, *J* = 8.1 Hz, 1H), 2.16 (m, 1H), 1.86 (m, 1H), 1.76 (m, 2H). ^13^C-NMR (150 MHz, CDCl_3_): δ 172.0, 138.7, 137.0, 133.3, 131.4, 128.7, 128.2, 127.0, 122.6, 120.6, 110.0, 67.3, 58.6, 53.3, 30.0, 23.4. HR–ESI–MS: *m*/*z* = 321.1706 [M + H]^+^, calcd. for C_19_H_21_N_4_O: 321.1710.

*(R)-1-Benzyl-N-(1H-indazol-5-yl)pyrrolidine-2-carboxamide* (**2b**): yield: 64%; white solid; [α]${}_{D}^{20}$ = +93 (c 0.23, MeOH); m.p.: 199–201 °C; ^1^H-NMR (400 MHz, CDCl_3_): δ 12.98 (s, 1H), 9.70 (s, 1H), 8.09 (s, 1H), 8.00 (s, 1H), 7.43 (m, 4H), 7.32 (t, *J* = 7.5 Hz, 2H), 7.21 (t, *J* = 6.9 Hz, 1H), 3.85 (d, *J* = 12.9 Hz, 1H), 3.58 (d, *J* = 12.9 Hz, 1H), 3.23 (dd, *J*_1_ = 4.8 Hz; *J*_2_ = 8.7 Hz, 1H), 3.02 (m, 1H), 2.33 (q, *J* = 8.1 Hz, 1H), 2.14 (m, 1H), 1.86 (m, 1H), 1.79 (m, 2H). ^13^C-NMR (150 MHz, CDCl_3_): δ 172.0, 138.7, 137.0, 133.2, 131.7, 131.4, 128.7, 128.2, 126.9, 122.6, 120.6, 109.9, 67.2, 58.6, 53.2, 29.9, 23.4. HR–ESI–MS: *m*/*z* = 321.1716 [M + H]^+^, calcd. for C_19_H_21_N_4_O: 321.1710.

*(S)-1-Benzyl-N-(1H-indazol-5-yl)pyrrolidine-2-carboxamide* (**2c**): yield: 59%; white solid; [α]${}_{D}^{20}$ = −95 (c 0.39, MeOH); m.p.: 202–203 °C; ^1^H-NMR (300 MHz, CDCl_3_): δ 12.95 (s, 1H), 9.67 (s, 1H), 8.08 (s, 1H), 7.99 (s, 1H), 7.40 (m, 4H), 7.30 (t, *J* = 7.2 Hz, 2H), 7.22 (d, *J* = 7.2 Hz, 1H), 3.85 (d, *J* = 12.9 Hz, 1H), 3.58 (d, *J* = 13.5 Hz, 1H), 3.23 (dd, *J*_1_ = 4.2 Hz; *J*_2_ = 8.7 Hz, 1H), 3.02 (m, 1H), 2.40 (q, *J* = 8.1 Hz, 1H), 2.16 (m, 1H), 1.86 (m, 1H), 1.77 (m, 2H). ^13^C-NMR (150 MHz, CDCl_3_): δ 172.0, 138.7, 136.9, 136.1, 133.63, 131.4, 128.7, 128.2, 126.9, 122.6, 120.6, 109.9, 67.2, 58.6, 53.2, 29.9, 23.4. HR–ESI–MS: *m*/*z* = 321.1717 [M + H]^+^, calcd. for C_19_H_21_N_4_O: 321.1710.

*N-(1H-Indazol-5-yl)-1-(4-methylbenzyl)pyrrolidine-2-carboxamide* (**2d**): yield: 51%; white solid; m.p.: 200–202 °C; ^1^H-NMR (400 MHz, CDCl_3_): δ 12.95 (s, 1H), 9.65 (s, 1H), 8.06 (s, 1H), 7.99 (s, 1H), 7.44 (q, *J* = 9.2 Hz, 2H), 7.27 (d, *J* = 7.6 Hz, 2H), 7.12 (d, *J* = 7.6 Hz, 2H), 3.80 (d, *J* = 13.2 Hz, 1H), 3.54 (d, *J* = 12.8 Hz, 1H), 3.21 (dd, *J*_1_ = 4.8 Hz; *J*_2_ = 8.8 Hz, 1H), 3.01 (m, 1H), 2.37 (q, *J* = 8.4 Hz, 1H), 2.23 (s, 3H), 2.13 (m, 1H), 1.84 (m, 1H), 1.75 (m, 2H). ^13^C-NMR (150 MHz, CDCl_3_): δ 172.1, 137.0, 136.0, 135.6, 133.3, 131.4, 128.8, 128.7, 122.6, 120.6, 109.9, 109.3, 66.2, 58.3, 53.2, 30.0, 23.4, 20.6. HR–ESI–MS: *m*/*z* = 335.1869 [M + H]^+^, calcd. for C_20_H_23_N_4_O: 335.1866.

*(R)-N-(1H-Indazol-5-yl)-1-(4-methylbenzyl)pyrrolidine-2-carboxamide* (**2e**): yield: 63%; white solid; [α]${}_{D}^{20}$ = +66 (c 0.7, MeOH); m.p.: 203–205 °C; ^1^H-NMR (400 MHz, CDCl_3_): δ 12.95 (s, 1H), 9.65 (s, 1H), 8.06 (s, 1H), 7.99 (s, 1H), 7.44 (q, *J* = 8.8 Hz, 2H), 7.27 (d, *J* = 7.6 Hz, 2H), 7.12 (d, *J* = 7.6 Hz, 2H), 3.80 (d, *J* = 12.8 Hz, 1H), 3.54 (d, *J* = 12.8 Hz, 1H), 3.21 (dd, *J*_1_ = 4.8 Hz; *J*_2_ = 9.2 Hz, 1H), 3.01 (m, 1H), 2.37 (q, *J* = 8.0 Hz, 1H), 2.23 (s, 3H), 2.14 (m, 1H), 1.83 (m, 1H), 1.75 (m, 2H). ^13^C-NMR (150 MHz, CDCl_3_): δ 172.0, 136.9, 136.0, 135.6, 133.3, 131.4, 128.8, 128.7, 122.6, 120.6, 109.9, 67.2, 58.3, 53.2, 29.9, 23.4, 20.6. HR–ESI–MS: *m*/*z* = 335.1869 [M + H]^+^, calcd. for C_20_H_23_N_4_O: 335.1866.

*(S)-N-(1H-Indazol-5-yl)-1-(4-methylbenzyl)pyrrolidine-2-carboxamide* (**2f**): yield: 56%; white solid; [α]${}_{D}^{20}$ = −69 (c 0.68, MeOH); m.p.: 204–206 °C; ^1^H-NMR (400 MHz, CDCl_3_): δ 12.95 (s, 1H), 9.65 (s, 1H), 8.06 (s, 1H), 7.99 (s, 1H), 7.44 (q, *J* = 9.2 Hz, 2H), 7.27 (d, *J* = 7.6 Hz, 2H), 7.11 (d, *J* = 8.0 Hz, 2H), 3.80 (d, *J* = 12.8 Hz, 1H), 3.54 (d, *J* = 13.2 Hz, 1H), 3.21 (dd, *J*_1_ = 4.8 Hz; *J*_2_ = 8.8 Hz, 1H), 3.02 (m, 1H), 2.37 (q, *J* = 8.4 Hz, 1H), 2.23 (s, 3H), 2.14 (m, 1H), 1.85 (m, 1H), 1.75 (m, 2H). ^13^C-NMR (150 MHz, CDCl_3_): δ 172.0, 136.9, 136.0, 135.6, 133.3, 131.4, 128.8, 128.7, 122.6, 120.6, 109.3, 66.7, 58.3, 53.1, 29.9, 23.4, 20.7. HR–ESI–MS: *m*/*z* = 335.1868 [M + H]^+^, calcd. for C_20_H_23_N_4_O: 335.1866.

*1-Benzyl-N-(1H-indazol-6-yl)pyrrolidine-2-carboxamide* (**3a**): yield: 57%; white solid; m.p.: 195–196 °C; ^1^H-NMR (400 MHz, CDCl_3_): δ 12.88 (s, 1H), 9.79 (s, 1H), 8.12 (s, 1H), 7.95 (s, 1H), 7.64 (d, *J* = 8.4 Hz, 1H), 7.39 (d, *J* = 7.6 Hz, 2H), 7.30 (t, *J* = 7.2 Hz, 2H), 7.20 (t, *J* = 7.2 Hz, 1H), 7.11 (d, *J* = 8.4 Hz, 1H), 3.84 (d, *J* = 13.2 Hz, 1H), 3.59 (d, *J* = 12.8 Hz, 1H), 3.26 (dd, *J*_1_ = 4.8 Hz; *J*_2_ = 9.2 Hz, 1H), 3.04 (m, 1H), 2.40 (q, *J* = 8.4 Hz, 1H), 2.17 (m, 1H), 1.83 (m, 1H), 1.76 (m, 2H). ^13^C-NMR (150 MHz, CDCl_3_): δ 172.4, 140.2, 138.7, 136.5, 133.2, 128.7, 128.2, 127.0, 120.4, 119.2, 114.4, 99.0, 67.3, 58.6, 53.3, 30.0, 23.4. HR–ESI–MS: *m*/*z* = 321.1713 [M + H]^+^, calcd. for C_19_H_21_N_4_O: 321.1710.

*(R)-1-Benzyl-N-(1H-indazol-6-yl)pyrrolidine-2-carboxamide* (**3b**): yield: 52%; white solid; [α]${}_{D}^{20}$ = +98 (c 0.26, MeOH); m.p.: 192–193 °C; ^1^H-NMR (400 MHz, CDCl_3_): δ 12.87 (s, 1H), 9.79 (s, 1H), 8.11 (s, 1H), 7.95 (s, 1H), 7.64 (d, *J* = 8.4 Hz, 1H), 7.39 (d, *J* = 7.6 Hz, 2H), 7.30 (t, *J* = 7.2 Hz, 2H), 7.20 (t, *J* = 7.2 Hz, 1H), 7.11 (d, *J* = 8.8 Hz, 1H), 3.84 (d, *J* = 13.2 Hz, 1H), 3.60 (d, *J* = 12.8 Hz, 1H), 3.26 (dd, *J*_1_ = 4.8 Hz; *J*_2_ = 9.2 Hz, 1H), 3.05 (m, 1H), 2.40 (q, *J* = 8.4 Hz, 1H), 2.17 (m, 1H), 1.83 (m, 1H), 1.76 (m, 2H). ^13^C-NMR (150 MHz, CDCl_3_): δ 172.4, 140.2, 138.7, 136.5, 133.2, 128.7, 128.2, 127.0, 120.5, 119.2, 114.4, 99.0, 67.3, 58.6, 53.3, 30.0, 23.4. HR–ESI–MS: *m*/*z* = 321.1713 [M + H]^+^, calcd. for C_19_H_21_N_4_O: 321.1710.

*(S)-1-Benzyl-N-(1H-indazol-6-yl)pyrrolidine-2-carboxamide* (**3c**): yield: 61%; white solid; [α]${}_{D}^{20}$ = −96 (c 0.49, MeOH); m.p.: 195–197 °C; ^1^H-NMR (400 MHz, CDCl_3_): δ 12.87 (s, 1H), 9.79 (s, 1H), 8.11 (s, 1H), 7.95 (s, 1H), 7.64 (d, *J* = 8.4 Hz, 1H), 7.39 (d, *J* = 7.2 Hz, 2H), 7.30 (t, *J* = 7.6 Hz, 2H), 7.20 (t, *J* = 7.2 Hz, 1H), 7.11 (d, *J* = 8.8 Hz, 1H), 3.84 (d, *J* = 12.8 Hz, 1H), 3.60 (d, *J* = 13.2 Hz, 1H), 3.26 (dd, *J*_1_ = 4.8 Hz; *J*_2_ = 9.2 Hz, 1H), 3.05 (m, 1H), 2.40 (q, *J* = 8.4 Hz, 1H), 2.17 (m, 1H), 1.83 (m, 1H), 1.76 (m, 2H). ^13^C-NMR (150 MHz, CDCl_3_): δ 172.4, 140.2, 138.7, 136.5, 133.2, 128.7, 128.2, 127.0, 120.5, 119.2, 114.4, 99.0, 67.3, 58.6, 53.3, 30.0, 23.4. HR–ESI–MS: *m*/*z* = 321.1715 [M + H]^+^, calcd. for C_19_H_21_N_4_O: 321.1710.

*1-Benzyl-N-(1H-indazol-5-yl)pyrrolidine-3-carboxamide* (**4a**): yield:42%; white solid; m.p.: 203–204 °C; ^1^H-NMR (300 MHz, DMSO-*d*_6_): δ 12.93 (s, 1H), 9.83 (s, 1H), 8.09 (s, 1H), 7.98 (s, 1H), 7.66 (d, *J* = 8.7 Hz, 1H), 7.38 (d, *J* = 9.0 Hz, 1H), 7.31 (d, *J* = 4.2 Hz, 4H), 7.24 (m, 1H), 3.59 (m, 2H), 3.05 (m, 1H), 2.88 (t, *J* = 8.7 Hz, 1H), 2.67 (m, 1H), 2.46 (m, 2H), 2.01 (m, 2H). ^13^C-NMR (150 MHz, DMSO-*d*_6_): δ 172.3, 158.1, 136.7, 133.2, 132.2, 129.7, 122.6, 120.3, 113.5, 109.9, 109.5, 58.5, 56.9, 54.9, 53.4, 43.4, 27.5. HR–ESI–MS: *m*/*z* = 321.17096 [M + H]^+^, calcd. for C_19_H_21_N_4_O: 321.17099.

*N-(1H-Indazol-5-yl)-1-(4-methylbenzyl)pyrrolidine-3-carboxamide* (**4b**): yield: 42%; white solid; ^1^H-NMR (400 MHz, DMSO-*d*_6_): δ 12.94 (s, 1H), 9.82 (s, 1H), 8.09 (s, 1H), 7.98 (s, 1H), 7.45 (d, *J* = 8.7 Hz, 1H), 7.38 (d, *J* = 8.4 Hz, 1H), 7.19 (d, *J* = 7.5 Hz, 2H), 7.11 (t, *J* = 7.8 Hz, 2H), 3.54 (s, 2H), 3.04 (m, 1H), 2.86 (t, *J* = 8.7 Hz, 1H), 2.66 (m, 1H), 2.42 (m, 2H), 2.28 (s, 3H), 2.01 (m, 2H). ^13^C-NMR (150 MHz, DMSO-*d*_6_): δ 172.3, 136.8, 135.8, 133.3, 132.3, 128.7, 128.5, 122.7, 120.3, 110.0, 109.5, 58.9, 57.0, 53.5, 43.4, 27.6, 20.7.

*N-(1H-Indazol-5-yl)-1-(4-methoxybenzyl)pyrrolidine-3-carboxamide* (**4c**): yield: 46%; white solid; ^1^H-NMR (400 MHz, DMSO-*d*_6_): δ 12.93 (s, 1H), 9.82 (s, 1H), 8.08 (s, 1H), 7.98 (s, 1H), 7.44 (d, *J* = 9.2 Hz, 1H), 7.38 (d, *J* = 8.8 Hz, 1H), 7.22 (d, *J* = 8.4 Hz, 2H), 6.86 (t, *J* = 8.4 Hz, 2H), 3.72 (s, 3H), 3.51 (d, *J* = 6.8 Hz, 2H), 3.03 (m, 1H), 2.85 (t, *J* = 8.7 Hz, 1H), 2.65 (m, 1H), 2.43 (m, 2H), 2.00 (m, 2H). ^13^C-NMR (150 MHz, DMSO-*d*_6_): δ 170.0, 159.6, 139.7, 133.3, 132.0, 131.8, 124.3, 122.6, 120.3, 114.0, 110.2, 109.8, 106.9, 56.4, 55.2, 54.4, 52.4, 42.3, 27.7. HR–ESI–MS: *m*/*z* = 351.18106 [M + H]^+^, calcd. for C_20_H_23_N_4_O_2_: 351.08155.

*1-(4-Bromobenzyl)-N-(1H-indazol-5-yl)pyrrolidine-3-carboxamide* (**4d**): yield: 62%; slightly yellow solid; m.p.: 235–236 °C; ^1^H-NMR (400 MHz, DMSO-*d*_6_): δ 12.93 (s, 1H), 9.82 (s, 1H), 8.08 (s, 1H), 7.98 (s, 1H), 7.50 (d, *J* = 8.0 Hz, 2H), 7.44 (d, *J* = 8.9 Hz, 1H), 7.38 (d, *J* = 9.0 Hz, 1H), 7.28 (d, *J* = 7.9 Hz, 2H), 3.56 (m, 2H), 3.05 (p, *J* = 7.7 Hz, 1H), 2.86 (t, *J* = 8.5 Hz, 1H), 2.67 (q, *J* = 7.4 Hz, 1H), 2.43 (m, 2H), 1.99 (m, 2H). ^13^C-NMR (150 MHz, DMSO-*d*_6_): δ 172.2, 138.7, 136.8, 133.3, 132.3, 131.0, 130.7, 122.7, 120.3, 119.8, 110.0, 109.6, 58.4, 57.0, 53.5, 43.5, 40.0, 27.7. HR–ESI–MS: *m*/*z* = 399.0808 [M + H]^+^, calcd. for C_19_H_20_ON_4_Br: 399.0815.

*N-(1H-Indazol-5-yl)-1-(4-methoxybenzyl)pyrrolidine-3-carboxamide* (**4e**): yield: 64%; slightly orange solid; m.p.: 197–199 °C; ^1^H-NMR (500 MHz, DMSO-*d*_6_): δ 12.93 (s, 1H), 9.84 (s, 1H), 8.19 (d, *J* = 8.4 Hz, 2H), 8.09 (s, 1H), 7.98 (s, 1H), 7.61 (d, *J* = 8.4 Hz, 2H), 7.50–7.33 (m, 2H), 3.74 (s, 2H), 3.07 (q, *J* = 7.9 Hz, 1H), 2.90 (t, *J* = 8.6 Hz, 1H), 2.71 (q, *J* = 7.6 Hz, 1H), 2.56 (t, *J* = 8.2 Hz, 1H), 2.03 (m, 2H). ^13^C-NMR (150 MHz, DMSO-*d*_6_): δ 172.2, 147.5, 146.5, 136.8, 133.3, 132.2, 129.5, 123.4, 122.7, 120.3, 110.0, 109.6, 58.3, 57.1, 53.7, 43.5, 27.8. HR–ESI–MS: *m*/*z* = 366.1548 [M + H]^+^, calcd. for C_19_H_20_N_5_O_3_: 366.1561.

*1-(4-Cyanobenzyl)-N-(1H-indazol-5-yl)pyrrolidine-3-carboxamide* (**4f**): yield: 61%; slightly yellow solid; m.p.: 202–204 °C; ^1^H-NMR (400 MHz, DMSO-*d*_6_): δ 12.93 (s, 1H), 9.83 (s, 1H), 8.09 (s, 1H), 7.98 (s, 1H), 7.79 (d, *J* = 7.8 Hz, 2H), 7.53 (d, *J* = 7.9 Hz, 2H), 7.49–7.32 (m, 2H), 3.69 (s, 2H), 3.07 (m, 1H), 2.87 (t, *J* = 8.5 Hz, 1H), 2.69 (m, 1H), 2.54 (m, 1H), 2.03 (m, 3H). ^13^C-NMR (150 MHz, DMSO-*d*_6_): δ 172.2, 145.3, 136.8, 133.3, 132.2, 132.2, 129.3, 122.7, 120.3, 118.9, 110.0, 109.6, 58.6, 57.1, 53.6, 43.5, 40.0, 27.8. HR–ESI–MS: *m*/*z* = 346.1641 [M + H]^+^, calcd. for C_20_H_20_N_5_O: 346.1662.

*1-(4-Fluorobenzyl)-N-(1H-indazol-5-yl)pyrrolidine-3-carboxamide* (**4g**): yield: 50%; white solid; m.p.: 202–203 °C; ^1^H-NMR (400 MHz, CDCl_3_): δ 12.93 (s, 1H), 9.82 (s, 1H), 8.09 (s, 1H), 7.98 (s, 1H), 7.44 (d, *J* = 8.8 Hz, 1H), 7.39 (s, 1H), 7.34 (t, *J* = 6.4 Hz, 2H), 7.13 (t, *J* = 8.8 Hz, 2H), 3.57 (d, *J* = 4.0 Hz, 2H), 3.04 (m, 1H), 2.86 (t, *J* = 8.4 Hz, 1H), 2.66 (m, 1H), 2.43 (m, 2H), 2.01 (m, 2H). ^13^C-NMR (150 MHz, DMSO-*d*_6_): δ 172.3, 162.1, 160.2, 136.8, 135.4, 133.3, 132.2, 130.3, 122.7, 120.3, 114.9, 114.7, 110.0, 109.6, 58.3, 57.0, 53.5, 27.6. HR–ESI–MS: *m*/*z* = 339.16138 [M + H]^+^, calcd. for C_19_H_20_N_4_OF: 339.16157.

*(S)-1-Benzyl-N-(1H-indazol-5-yl)pyrrolidine-3-carboxamide* (**4h**): yield: 53%; white solid; [α]${}_{D}^{18}$ = +26 (c 1.05, MeOH); m.p.: 203–204 °C; ^1^H-NMR (300 MHz, CDCl_3_): δ 12.93 (s, 1H), 9.83 (s, 1H), 8.09 (s, 1H), 7.98 (s, 1H), 7.66 (d, *J* = 8.7 Hz, 1H), 7.38 (d, *J* = 9.0 Hz, 1H), 7.31 (d, *J* = 4.2 Hz, 4H), 7.24 (m, 1H), 3.59 (s, 1H), 3.05 (m, 1H), 2.88 (t, *J* = 8.7 Hz, 1H), 2.67 (m, 1H), 2.46 (m, 2H), 2.01 (m, 2H).

*(R)-1-Benzyl-N-(1H-indazol-5-yl)pyrrolidine-3-carboxamide* (**4i**): yield: 53%; white solid; [α]${}_{D}^{18}$ = −25 (c 1.0, MeOH); m.p.: 203–204 °C; ^1^H-NMR (300 MHz, CDCl_3_): δ 12.93 (s, 1H), 9.83 (s, 1H), 8.09 (s, 1H), 7.98 (s, 1H), 7.66 (d, *J* = 8.7 Hz, 1H), 7.38 (d, *J* = 9.0 Hz, 1H), 7.31 (d, *J* = 4.2 Hz, 4H), 7.24 (m, 1H), 3.59 (s, 1H), 3.05 (m, 1H), 2.88 (t, *J* = 8.7 Hz, 1H), 2.67 (m, 1H), 2.46 (m, 2H), 2.01 (m, 2H).

*1-Benzoyl-N-(1H-indazol-5-yl)pyrrolidine-3-carboxamide* (**5a**): yield: 77%; yellow solid; m.p.: 211–213 °C; ^1^H-NMR (500 MHz, DMSO-*d*_6_): δ 12.96 (s, *J* = 9.5 Hz, 1H), 10.08 (s, 0.5H), 9.96 (s, 0.5H), 8.14 (s, 0.5H), 8.07 (s, 0.5H), 7.99 (d, *J* = 14.5 Hz, 1H), 7.52 (m, 2H), 7.48–7.35 (m, 5H), 3.83–3.45 (m, 4H), 3.28–3.09 (m, 1H), 2.13 (m, 2H). ^13^C-NMR (150 MHz, DMSO-*d*_6_): δ 170.7, 170.1, 168.2, 168.1, 136.9, 136.9, 133.3, 132.0, 131.9, 129.8, 128.2, 128.2, 127.0, 122.6, 120.3, 120.3, 110.1, 109.8, 109.8, 51.4, 48.6, 45.5, 44.7, 42.9, 30.1, 27.9. HR–ESI–MS: *m*/*z* = 335.1486 [M + H]^+^, calcd. for C_19_H_19_N_4_O_2_: 335.1503.

*N-(1H-Indazol-5-yl)-1-(4-methylbenzoyl)pyrrolidine-3-carboxamide* (**5b**): yield: 78%; yellow solid; m.p.: 224–226 °C; ^1^H-NMR (400 MHz, DMSO-*d*_6_): δ 12.95 (s, 1H), 10.08 (s, 0.5H), 9.97 (s, 0.5H),8.14 (s, 0.5H), 8.07 (s, 0.5H), 7.99 (d, *J* = 10.0 Hz, 1H), 7.55–7.32 (m, 4H), 7.24 (d, *J* = 7.5 Hz, 2H), 3.84–3.44 (m, 4H), 3.18 (m, 1H), 2.33 (s, 3H), 2.24–1.94 (m, 2H). ^13^C-NMR (150 MHz, DMSO-*d*_6_): δ 170.8, 170.1, 168.3, 168.1, 139.5, 139.5, 136.8, 134.0, 133.9, 133.3, 132.1, 132.0, 129.6, 128.7, 127.2, 122.6, 120.3, 120.2, 110.1, 109.8, 109.7, 51.5, 48.7, 48.7, 45.5, 44.7, 42.9, 35.1, 31.3, 30.2, 29.1, 29.0, 29.0, 28.9, 28.8, 28.7, 28.7, 28.6, 27.9, 26.5, 25.1, 22.1, 20.9, 13.9. HR–ESI–MS: *m*/*z* = 349.1639 [M + H]^+^, calcd. for C_20_H_21_N_4_O_2_: 349.1659.

*N-(1H-Indazol-5-yl)-1-(4-methoxybenzoyl)pyrrolidine-3-carboxamide* (**5c**): yield: 79%; white solid; m.p.: 242–244 °C; ^1^H-NMR (400 MHz, DMSO-*d*_6_): δ 12.95 (s, 1H), 10.07 (s, 0.5H), 9.95 (s, 0.5H), 8.13 (s, 0.5H), 8.07 (s, 0.5H), 7.99 (m, 1H), 7.52 (d, *J* = 8.3 Hz, 2H), 7.49–7.32 (m, 2H), 6.97 (d, *J* = 8.3 Hz, 2H), 3.79 (s, 3H), 3.73–3.45 (m, 4H), 3.17 (m, 1H), 2.14 (m, 2H). ^13^C-NMR (150 MHz, DMSO-*d*_6_): δ 170.8, 170.1, 168.0, 167.8, 160.4, 136.8, 133.4, 132.0, 129.2, 128.8, 122.7, 120.3, 120.3, 113.4, 110.1, 109.8, 109.7, 55.2, 51.6, 48.8, 45.7, 44.8, 42.9, 30.2, 27.9. HR–ESI–MS: *m*/*z* = 365.1590 [M + H]^+^, calcd. for C_20_H_21_N_4_O_3_: 365.1608.

*1-(4-Bromobenzoyl)-N-(1H-indazol-5-yl)pyrrolidine-3-carboxamide* (**5d**): yield: 63%; slightly orange solid; m.p.: 250–251 °C; ^1^H-NMR (500 MHz, DMSO-*d*_6_): δ 12.95 (s, 1H), 10.08 (s, 0.5H), 9.96 (s, 0.5H), 8.13 (s, 0.5H), 8.07 (s, 0.5H), 7.99 (d, *J* = 11.7 Hz, 1H), 7.64 (d, *J* = 8.1 Hz, 2H), 7.52–7.32 (m, 4H), 3.85–3.43 (m, 4H), 3.19 (m, 1H), 2.31–1.98 (m, 2H). ^13^C-NMR (150 MHz, DMSO-*d*_6_): δ 170.7, 170.1, 167.2, 167.1, 136.9, 136.0, 135.9, 133.2, 132.0, 131.9, 131.3, 131.2, 129.3, 123.3, 123.2, 122.6, 120.3, 120.3, 110.2, 109.8, 109.8, 51.3, 48.7, 48.6, 45.6, 44.7, 42.9, 30.2, 27.9. HR–ESI–MS: *m*/*z* = 413.0591 [M + H]^+^, calcd. for C_19_H_18_N_4_O_2_Br: 413.0608.

*N-(1H-Indazol-5-yl)-1-(4-nitrobenzoyl)pyrrolidine-3-carboxamide* (**5e**): yield: 78%; yellow solid; m.p.: 177–178 °C; ^1^H-NMR (500 MHz, DMSO-*d*_6_): δ 13.00 (d, *J* = 9.8 Hz, 1H), 10.29 (d, *J* = 6.0 Hz, 1H), 10.18 (d, *J* = 6.5 Hz, 1H), 8.27 (d, *J* = 8.3 Hz, 2H), 8.16 (s, 0.5H), 8.09 (s, 0.5H), 7.99 (d, *J* = 14.3 Hz, 1H), 7.79 (dd, *J* = 8.3, 6.1 Hz, 2H), 7.48 (s, 1H), 7.43 (t, *J* = 8.7 Hz, 1H), 3.87–3.40 (m, 4H), 3.32–3.19 (m, 1H), 2.34–2.01 (m, 2H). ^13^C-NMR (150 MHz, DMSO-*d*_6_): δ 170.7, 170.1, 166.4, 166.3, 147.9, 147.9, 142.9, 142.9, 136.9, 136.8, 133.3, 133.3, 132.2, 132.1, 128.5, 123.6, 123.6, 122.6, 122.6, 120.3, 120.2, 110.1, 110.0, 109.7, 109.7, 51.2, 48.7, 48.4, 45.6, 44.6, 42.8, 30.1, 28.0. HR–ESI–MS: *m*/*z* = 380.1338 [M + H]^+^, calcd. for C_19_H_18_N_5_O_4_: 380.1353.

*1-(4-Chlorobenzoyl)-N-(1H-indazol-5-yl)pyrrolidine-3-carboxamide* (**5f**): yield: 77%; white solid; m.p.: 225–227 °C; ^1^H-NMR (400 MHz, DMSO-*d*_6_): δ 12.95 (s, 1H), 10.09 (s, 0.5H), 9.97 (s, 0.5H), 8.14 (s, 0.5H), 8.07 (s, 0.5H), 8.00 (d, *J* = 9.4 Hz, 1H), 7.68–7.28 (m, 6H), 3.88–3.55 (m, 3H), 3.55–3.46 (m, 1H), 3.19 (m, 1H), 2.26–2.00 (m, 2H). ^13^C-NMR (150 MHz, DMSO-*d*_6_): δ 170.7, 170.0, 167.1, 167.0, 136.9, 136.8, 135.6, 135.6, 134.5, 134.5, 133.4, 133.3, 132.0, 131.9, 129.1, 128.3, 128.3, 122.7, 122.6, 120.3, 120.3, 110.1, 110.1, 109.8, 109.8, 51.3, 48.7, 48.6, 45.6, 44.7, 42.9, 30.2, 27.9. HR–ESI–MS: *m*/*z* = 369.1101 [M + H]^+^, calcd. for C_19_H_18_N_4_O_2_Cl: 369.1113.

### 3.2. Bioassay Studies

#### 3.2.1. Rho Kinase Activity Assay

Rho kinase assay kit (CY-1160, Cyclex, Nagoya, Japan) was employed to detect the inhibitory effect of the compounds on ROCK I following the manufacturer’s instructions. Briefly, the compounds at 20 μM were pre-incubated in a system wherein ROCK I (0.02 ng/μL) phosphorylates the kinase substrate’s myosin-binding subunit (MBS) pre-absorbed onto the microplate in the presence of Mg^2+^ and ATP. Then, the system was washed after incubation at 30 °C for 30 min. 100 μL of horseradish peroxidase-conjugated anti-AF20 antibody was added to the wells and then incubated for 30 min at room temperature. The reaction system was then incubated with the substrate tetra-methylbenzidine (TMB), which can be catalyzed from a colorless solution to a blue solution. The absorbance was measured at a wavelength of 450 nm on a microplate reader. The color reflects the relative level of ROCK I activity. The inhibitory rates of the compounds on ROCK I were obtained from OD 450 nm and the IC_50_ values were calculated by nonlinear regression.

#### 3.2.2. Vasorelaxant Activity Test

Male Sprague-Dawley rats weighing 240–280 g (Vital River Laboratories, Beijing, China) were maintained in a 12-h light/dark cycle at 24 °C in a relative humidity of 60% room, and received food and water ad libitum. The animal care and handling were performed in accordance with the Guide for the Care and Use of Laboratory Animals published by the US National Institutes of Health and the Laboratories Institutional Animal Care and Use Committee of the Chinese Academy of Medical Science and Peking Union Medical College (ethic approval number: 00001012).

The vasorelaxant activity assay was performed as described previously [35]. Briefly, the descending thoracic aorta was isolated after rats were euthanized by cervical dislocation. Then, the aorta was cut into ring segments (3–4 mm in length). The endothelial layer of the aorta was destroyed by gently rubbing the luminal surface with a moist cotton swab when necessary. Two stainless-steel triangles were inserted into the lumen of each ring. One triangle was fixed, and the other attached to a force transducer. Changes in isometric force were recorded on a BIOPAC polygraph (MP100WSW, Biopac Systems, Inc, Goleta, CA, USA). The rings were mounted in organ baths containing 10 mL Krebs-Henseleit (K-H) buffer with the following composition (mM): NaCl 120, KCl 4.8, MgSO_4_ 1.4, KH_2_PO_4_ 1.2, glucose 11.0, CaCl_2_ 2.5, NaHCO_3_ 25.0 and EDTA 0.01. The K-H buffer was continuously bubbled with 95% O_2_/5% CO_2_ at 37 °C. Rings were allowed to equilibrate for 60 min at a resting tension of 1.2 g with changes of buffer every 20 min.

After the equilibration period, the aortic rings were constricted with a high K^+^ (60 mM) K-H solution to stimulate the tissue and to test its availability. Then, the rings were washed with normal K-H buffer to restore the basic tension of 1.2 g. The aortic rings were then stimulated with a high K^+^ (60 mmol/L) K-H buffer ornorepinephrine (1 μM) to evaluate the vasorelaxant effects of the compounds. When the contraction reached the platform, compounds were added to the bath in batches at 5-min intervals to reach the accumulative concentrations of 1, 2, 5, 10, 25 and 50 μM. The tension of vessels was recorded and nonlinear regression was used to calculate the EC_50_ values.

### 3.3. Molecular Docking

Molecular docking was performed with Discovery Studio 2016 software package (version 2016, BIOVIA, San Diego: *Dassault Systèmes*, CA, USA, 2016). The crystal structure of ROCK I, with co-crystal ligand obtained from the Protein Data Bank (PDB code: 3NDM) [36], was used to simulate the binding mode between our compounds and ROCK I protein. The original water molecules were removed from the coordinates set. The co-crystal ligand was used to determine the binding site and was then removed prior to docking. The docking calculation was carried out by using the LibDock protocol. Smart Minimizer algorithm was used to minimize docked poses with CHARMm force field. The default parameter settings were used. The obtained docking poses were ranked by LibDock score and the best-scored pose was chosen.

## 4. Conclusions

In summary, a series of potent ROCK I inhibitors based on a hybrid prolinamido indazole scaffold were designed and synthesized. Six compounds **4a**, **4b**, **4c**, **4d**, **4g** and **4h** showed activity profiles superior to DL0805, validating our design strategy. These results have established preliminary SAR trends, and molecular docking studies on the active compound **4a** provide a foundation for future molecular optimization of this promising scaffold.

## Figures and Tables

**Table 1:** Percentage inhibition of **2a**–**2f**, **3a**–**3c**, **4a**–**4i** and **5a**–**5f** against ROCK I under the concentration of 20μM. ^a^

Compound	R	Configuration	% Inhibition of ROCK I	Compound	R	Configuration	% Inhibition of ROCK I
**2a**	4-H	(±)	27.2	**4e**	4-NO_2_	(±)	44.7
**2b**	4-H	(*R*)	24.3	**4f**	4-CN	(±)	24.5
**2c**	4-H	(*S*)	24.6	**4g**	4-F	(±)	63.8
**2d**	4-CH_3_	(±)	26.6	**4h**	4-H	(*S*)	54.8
**2e**	4-CH_3_	(*R*)		**4i**	4-H	(*R*)	73.9
**2f**	4-CH_3_	(*S*)	21.3	**5a**	4-H	(±)	42.5
**3a**	4-H	(±)	16.0	**5b**	4-CH_3_	(±)	54.9
**3b**	4-H	(*R*)	15.0	**5c**	4-OCH_3_	(±)	19.1
**3c**	4-H	(*S*)	23.7	**5d**	4-Br	(±)	27.9
**4a**	4-H	(±)	73.2	**5e**	4-NO_2_	(±)	45.4
**4b**	4-CH_3_	(±)	75.8	**5f**	4-Cl	(±)	55.2
**4c**	4-OCH_3_	(±)	53.3	DL0805			72.3
**4d**	4-Br	(±)	62.5	fasudil			71.1

^a^ These data were obtained by single determinations.

**Table 2:** The IC_50_ values of compounds against ROCK I.

Compound	ROCK I (μM)	Compound	ROCK I (μM)	Compound	ROCK I (μM)
**4a**	0.27 ^a^	**4g**	1.41 ^b^	**5f**	7.26 ^b^
**4b**	0.17 ^a^	**4h**	0.42 ^b^	DL0805	6.67 ^b^
**4c**	1.86 ^b^	**4i**	7.32 ^b^	fasudil	0.36 ^a^
**4d**	0.51 ^b^	**5b**	11.5 ^b^		

^a^ These data were means of multiple experiments (*N* = 4) with errors within 30% of the mean; ^b^ these data were obtained by single determinations.

**Table 3:** The EC_50_ values of compounds for vasorelaxant activity.

Compound	Vasorelexant Evaluation (μM)	
	In High-Potassium Model	In NE Model
**4a**	4.00 ^a^	5.62 ^a^
**4b**	3.47 ^a^	4.07 ^a^
**4c**	5.80 ^a^	5.57 ^a^
**4d**	58.1 ^b^	46.8 ^b^
**4g**	19.5 ^b^	8.91 ^b^
**4h**	32.36 ^b^	--
**4i**	6.02 ^b^	11.48 ^b^
**5b**	60.6 ^b^	15.6 ^b^
**5f**	--	--
**DL0805**	9.44 ^b^	28.8 ^b^
**fasudil**	5.47 ^a^	4.65 ^a^

-- No activity; ^a^ these data were means of multiple experiments (*N* = 6) with errors within 30% of the mean; ^b^ these data were obtained by single determinations.
